# Editorial: Peripheral Markers of Immune Response in Major Psychiatric Disorders: Where Are We Now and Where Do We Want to Be?

**DOI:** 10.3389/fpsyt.2019.00005

**Published:** 2019-01-22

**Authors:** Błazej Misiak, Dorota Frydecka, Bartłomiej Stanczykiewicz, Jerzy Samochowiec

**Affiliations:** ^1^Department of Genetics, Wroclaw Medical University, Wrocław, Poland; ^2^Department of Psychiatry, Wroclaw Medical University, Wrocław, Poland; ^3^Department of Nervous System Diseases, Wroclaw Medical University, Wrocław, Poland; ^4^Department of Psychiatry, Pomeranian Medical University, Szczecin, Poland

**Keywords:** immunity, schizophrenia, depression, bipolar disoder, inflammation

Psychiatric disorders represent complex phenotypes with imprecise diagnostic boundaries. Shifting the paradigm of understanding this complexity from clinical assessment to biological operationalization is a far goal of studies investigating the pathophysiology of mental disorders. Immune-inflammatory alterations observed in mental disorders hold a great promise for better understanding disease etiopathology, development of novel treatment strategies, and improvement of clinical outcomes. Indeed, psychiatric disorders, especially schizophrenia, bipolar disorder, and major depression, are characterized by several immune-inflammatory alterations outside the brain, including elevated levels of pro-inflammatory cytokines, specific and non-specific autoantibodies and acute phase proteins, as well as abnormal counts of lymphocyte subpopulations. Some immune-inflammatory alterations represent state markers that occur in illness exacerbation and normalize with pharmacological treatment, whereas other disturbances serve as trait markers that are present regardless of illness' stage (1). Although overlapping dysregulations of immune-inflammatory response can be observed in major psychiatric disorders, certain differences can be also found and might improve diagnostic management strategies (2). In this research topic we provide a forum of new perspectives, emerging concepts, and novel immune-inflammatory disturbances in psychiatric disorders.

Causal mechanisms of immune-inflammatory alterations remain unknown. In their review article, Rudzki and Szulc raise a timely question whether immune-inflammatory alterations in mental disorders appear due to changes in the gut microbiota or the “leaky gut” phenomenon. The authors review various mechanisms underlying aberrant performance of the gut-brain axis in major psychiatric disorders, with special emphasis on schizophrenia, bipolar disorder, and major depression. They conclude that this field of studies should open the new era of clinical trials investigating the efficacy of interventions that aim to restore the gut microbial homeostasis in mental disorders.

Another concept of causality was presented by Ratajczak et al. Indeed, the authors postulate that the sterile inflammation of the brain may trigger the onset of psychiatric disorders. According to the authors, sterile inflammation is initiated by the mannan-binding lectin pathway of the complement cascade activation. Interestingly, another study by Reginia et al. published in frame of our research topic, for the first time investigated complement cascade components in patients with bipolar disorder. This study revealed elevated levels of C3a and C5a components in patients with bipolar disorder compared to healthy controls. In turn, the levels of C5b-9 were significantly higher in patients with bipolar II disorder compared to those with bipolar I disorder.

Novel markers of immune activation in psychiatric disorders were also investigated by Borovcanin et al. The authors determined serum levels of innate immunity mediators—interleukin(IL)-33 and its receptor (ST2) together with galectin-3 in patients with schizophrenia. The levels of IL-33 and ST2 were significantly higher in patients with psychotic exacerbation compared to those in remission and healthy controls. Moreover, there were significant positive correlations between the levels of IL-33 and a number of positive and general psychopathology symptoms. Activation of innate immunity is increasingly being recognized as one of crucial aspects of aberrant immune response in schizophrenia (3–5).

In four review articles, the role of known markers of immune activation in psychiatric disorders was reviewed. Teixeira et al. focused on the role of eotaxin-1 (CCL11) in the pathophysiology of schizophrenia, bipolar disorder and major depression. Eotaxin-1 is a chemokine involved in selective recruitment of eosinophils to the sites of inflammation. It also plays an important role in aging, neurogenesis, and neurodegeneration. A recent meta-analysis of chemokine alterations in patients with schizophrenia revealed elevated levels of eotaxin-1 in multiple-episode schizophrenia patients but not in first-episode psychosis patients (6). These findings suggest that elevated eotaxin-1 level might be the marker of schizophrenia progression or might reflect medication effects. In turn, Borovcanin et al. reviewed the relevance of IL-6 to the pathophysiology of schizophrenia. The authors highlighted the role of this cytokine in illness progression, cognitive impairment and metabolic dysregulation. Fond et al. provided an updated systematic review of studies investigating the levels of C-reactive protein (CRP) in patients with schizophrenia. The authors concluded that elevated CRP levels in patients with schizophrenia might be related to cognitive impairment, hypovitaminosis D, microbiota alterations, nicotine dependence, and comorbid metabolic syndrome. Finally, Ohnuma et al. summarized results of their studies, regarding carbonyl stress and microinflammation in patients with schizophrenia, highlighting their potential relevance as markers of clinical outcomes.

This research topic provides novel insights from original studies, investigating known markers of inflammation in psychiatric disorders. van den Ameele et al. studied the levels of CRP, IL-6, tumor necrosis factor-α (TNF-α), and interferon-γ (IFN-γ) together with the levels of monoamine metabolism markers (neopterin, tryptophan, kynurenine, phenylalanine, and tryptophan) in patients with bipolar disorder and healthy controls. In the group of patients, but not in controls, older age was associated with increases in the levels of IL-6, CRP, neopterin, and the kynurenine/tryptophan ratio. These results point to the concept of accelerated aging in patients with bipolar disorder (7). In turn, Atake et al. investigated serum levels of brain-derived neurotrophic factor (BDNF) with respect to cognitive performance in chronic schizophrenia patients. Importantly, BDNF not only plays a role in neurogenesis, but also modulates immune-inflammatory responses (8). The authors found that aging, higher dosage of antipsychotics and anticholinergics as well as lower levels of BDNF are related to worse cognitive performance in patients with chronic schizophrenia.

Subclinical inflammation is traditionally perceived as the phenomenon, appearing in schizophrenia, bipolar disorder, and major depression. Wang et al. in their review article, indicate that Posttraumatic Stress Disorder (PTSD) is also associated with aberrant immune-inflammatory responses. The authors suggest that immune-inflammatory alterations in PTSD might have important clinical implications. They provide evidence that subclinical inflammation following traumatic events might predict the development of PTSD. Moreover, higher levels of pro-inflammatory markers might be associated with the development of comorbid physical health impairments in patients with PTSD.

Translation of findings from studies, investigating immune-inflammatory responses in psychiatric disorders, into clinical practice is a far goal of research activity in this field. Firstly, we still do not know whether subclinical inflammation is causally related to the development of mental disorders or simply represents a downstream effector. Investigating alterations of gut microbiota and compromised intestinal permeability is one of perspectives toward disentangling this conundrum. Longitudinal studies of subclinical inflammation in patients at early stages of illness with respect to clinical outcomes might also improve our knowledge regarding causality. Dissecting differences in immune-inflammatory alterations across various psychiatric disorders via implementation of high-throughput technologies, instead of studying single markers, should also be highlighted as one of most important future directions. These approaches are needed before final conclusions regarding relevance of immune-inflammatory alterations as diagnostic tools and potential treatment targets will be established.

## Author Contributions

BM wrote the first draft of the manuscript. DF, BS, and JS provided critical revision of the manuscript and important intellectual contributions. All authors read and approved the submitted version.

### Conflict of Interest Statement

The authors declare that the research was conducted in the absence of any commercial or financial relationships that could be construed as a potential conflict of interest.
